# Effectiveness of 23-valent pneumococcal polysaccharide vaccine on elderly patients with colorectal cancer: A population-based propensity score–matched cohort study

**DOI:** 10.1097/MD.0000000000018380

**Published:** 2019-12-16

**Authors:** Wen-Yen Chiou, Shih-Kai Hung, Hon-Yi Lin, Liang-Cheng Chen, Feng-Chun Hsu, Shiang-Jiun Tsai, Ben-Hui Yu, Moon-Sing Lee, Chung-Yi Li

**Affiliations:** a Department of Radiation Oncology, Dalin Tzu Chi Hospital, Buddhist Tzu Chi Medical Foundation, Chiayi; b School of Medicine, Tzu Chi University, Hualien; c Department of Public Health, College of Medicine, National Cheng Kung University, Tainan; d Department of Public Health, China Medical University, Taichung, Taiwan.

**Keywords:** colorectal cancer, pneumococcal polysaccharide vaccine, pneumonia

## Abstract

The commonly used vaccine for adults with a high risk of pneumonia is 23-valent pneumococcal polysaccharide vaccine (PPSV23). However, its effectiveness in patients with colorectal cancer has not been investigated. This study aimed to investigate the effectiveness of PPSV23 in reducing the risk of pneumonia among elderly patients with colorectal cancer.

A total of 120,605 newly diagnosed patients with colorectal cancer were identified from the Taiwan National Health Insurance Research Database between 1996 and 2010. Of these patients, 18,468 were 75 years or older in 2007 to 2010, and 3515 received PPSV23. People aged 75 years or older have been considered eligible for receiving PPSV23 vaccination in Taiwan since 2007. The specific “vaccination period” of October 2008 to December 2008 was used to minimize the potential immortal time bias. Therefore, 893 patients who received PPSV23 outside this vaccination period or died before 2009 and 2960 unvaccinated patients who died before 2009 were excluded. After the propensity score was matched with a 1:3 ratio, 2622 vaccinated patients and 7866 unvaccinated patients were recruited. A multivariate log-linear Poisson regression model was performed and adjusted for potential confounders, including influenza vaccination, vaccination period, cancer treatment modalities, comorbidities, and sociodemographic variables.

After 2 years of follow-up, the incidence rate of the pneumonia hospitalization of the vaccinated patients was significantly lower than that of the unvaccinated patients at 85.53 per 1000 person-years (PYs) of the former and 92.38 per 1000 PYs of the latter. The proportions of patients who had 2, 3, and >3 pneumonia hospitalizations per year were consistently lower in the vaccinated group than in the unvaccinated group (1.9% vs 2.0%, 0.5% vs 0.9%, and 0.7% vs 1.1%, respectively). After adjustment for covariates was made, PPSV23 vaccine was significantly associated with a reduced risk of pneumonia hospitalization, with an adjusted incidence rate ratio of 0.88 (*P* = .040). The overall pneumonia-free survival rate was also significantly higher in the vaccinated patients than in the unvaccinated patients (*P* = .001).

PPSV23 vaccination was associated with a significantly reduced rate of pneumonia hospitalization in elderly patients with colorectal cancer.

## 1 Introduction

Colorectal cancer (CRC) is the third most commonly diagnosed malignancy and the fourth leading cause of cancer deaths in the world; it accounted for about 1.4 million new cases and almost 700,000 deaths in 2012.^[1]^ It is also one of the leading causes of cancer-related deaths in the USA, Europe, and Asia.^[2]^ Taiwan has a high human development index, 0.907 in 2018. Similar to many other developed countries, CRC is a major public health problem in Taiwan. According to a report of the Bureau of Health Promotion, Taiwan, CRC has been considered the most common malignancy in Taiwan since 2006, and its crude incidence rate was 65.84 per 100,000 people in 2015.^[3]^ The standardized incidence rates of colon cancer and rectal cancer were 26.96 and 15.74 per 100,000 people in 2015 in Taiwan, respectively.^[3]^

Cancer treatment modalities, such as surgery, radiotherapy, chemotherapy, and targeted therapies can impair the immune system and increase susceptibility to pneumonia.^[4–6]^ Pneumonia is the most frequent type of infection in patients with cancer, and it is associated with high mortality rates.^[7]^ In a German cohort of 89,007 patients with cancer, the standardized incidence rate of pneumonia increases by 21-fold (lung cancer), 4.3-fold (hematological malignancies), and 1.8-fold (gastrointestinal tract malignancies) to 1.7-fold (breast cancer) compared with that of the matched control cohort.^[8]^ Schmedt et al^[8]^ also reported that 30-day mortality in community-acquired pneumonia (CAP) cases is the highest in patients with lung cancer (20.0%), and this parameter ranges from 7.2% to 18.5% in CAP cases compared with other cancer subtypes. Pneumonia can increase mortality, number and severity of complications, length of hospitalization, and hospital-related costs in patients with cancer.^[9]^

Among different pathogens causing pneumonia, *Streptococcus pneumoniae* is an important pathogen and still a major cause of morbidity and mortality worldwide.^[10]^ Invasive pneumococcal disease among healthy adults is effectively prevented by 23-valent pneumococcal polysaccharide vaccine (PPSV23; 50%–85%), which was licensed in 1983.^[11,12]^ The effectiveness of PPSV23 has, however, never been studied in patients with CRC.

Anticancer therapies may affect immune responses to vaccination, and their ability to prevent the development of an adequate immune response to influenza or pneumococcal pneumonia vaccine remains controversial. A previous study showed that serum antibody response to influenza virus vaccine in patients receiving cancer chemotherapy is weak. Some studies have, however, demonstrated that pneumococcal vaccine can stimulate an adequate immune antibody response in patients with nonspecific cancer.^[13–15]^ Another study also showed that the seroconversion rate of patients with CRC and receiving chemotherapy (36%) is lower than that of healthy volunteers without CRC (85%; *P* = .027).^[16]^ For clinical effectiveness, no clinical follow-up studies on patients with CRC have been performed.

Aging is another factor affecting the immune system. Age-dependent changes are referred to as immunosenescence, and they are partially responsible for poor immune responses to infections and low efficacy of vaccination in elderly persons.^[17]^

In this study, we investigated the effectiveness of PPSV23 in elderly patients with CRC and aged 75 years or older.

## 2 Materials and methods

Our retrospective cohort study involved a specific “vaccination period” from October 2008 to December 2008. Data, including comorbidities, were obtained from 1996 to 2010.

### 2.1 Sources of data and ethics statement

Data were obtained from the National Health Insurance Research Database (NHIRD) and released for research purposes by the National Health Research Institutes, Taiwan. The NHIRD contains medical claims data for approximately 99% of Taiwanese people.^[18]^ This study was done in accordance with the Helsinki Declaration and approved by the institution review board (IRB) of our institution, that is, Dalin Tzu Chi Hospital of Buddhist Tzu Chi Medical Foundation (approval number, B10404001). The IRB waived the requirement for written informed consents from the patients involved because the researchers could not directly contact individual patients from this de-identified database.

To ensure the accuracy of the claims, the National Health Insurance Administration (NHIA) performs quarterly expert reviews on every 50 to 100 ambulatory and inpatient claims filed by each medical institution.^[19]^ False diagnostic reports are liable to severe penalties from the NHIA.^[20]^

All claims data of patients with cancer between 1996 and 2010 were used. The databases contained ambulatory care claims, inpatient hospitalization claims, national cancer registration database, registry of catastrophic illness, and registry of beneficiaries, which recorded an individual's monthly income data. In Taiwan, the NHIA issues catastrophic illness certificates to all patients with pathologically confirmed malignant tumors.

### 2.2 Patients and study groups

**Figure 1:**
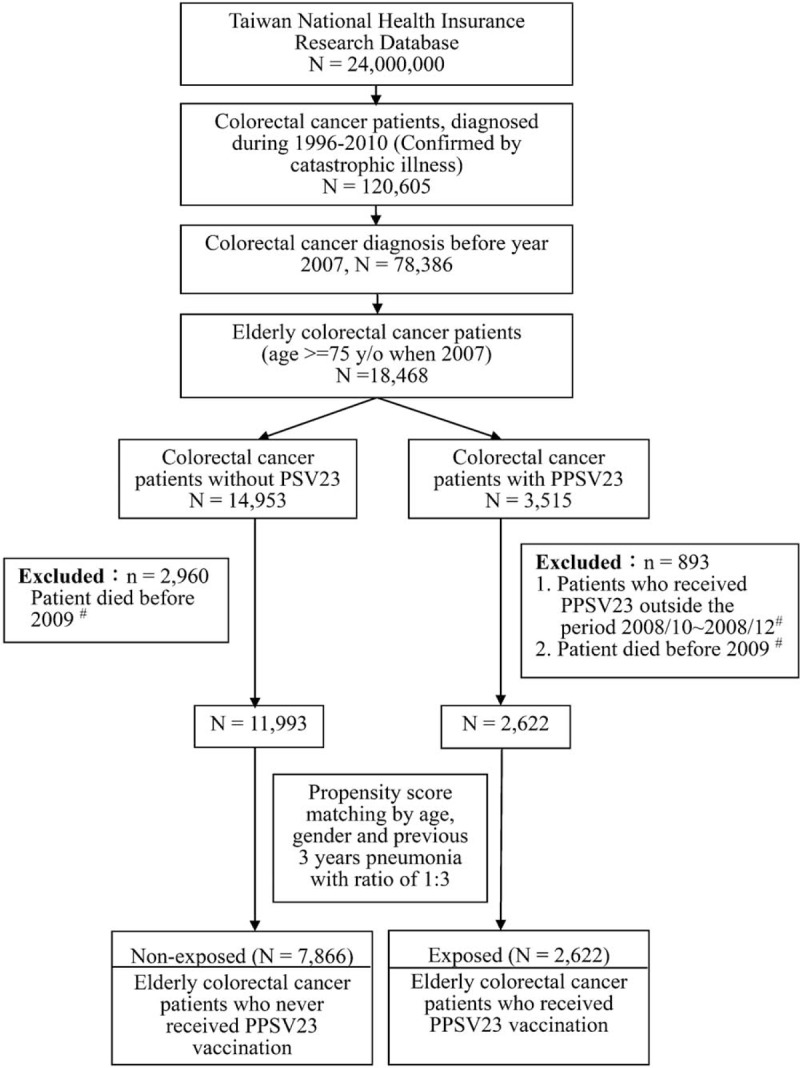
Study design flowchart for elderly colorectal cancer patients with and without 23-valent pneumococcal polysaccharide vaccine (PPSV23). ^#^ The “vaccination period,” October 2008 to December 2008, is set to reduce immortal time bias.

A total of 120,605 patients with CRC were identified from the national cancer registration database and validated by the information from the catastrophic illness registry. In Taiwan, the policy of administering PPSV23 free of charge for people aged 75 years or older started in 2007. In this study, the effectiveness of vaccine for patients with CRC and receiving vaccine after cancer diagnosis was explored. Therefore, only patients who had CRC diagnosed before 2007 were included. Our study subjects were limited to those aged older than 75 years. The flowchart of the study subjects’ enrollment is presented in Figure 1.

A total of 18,468 elderly patients with CRC diagnosed before 2007 were included. Among them, 3515 received PPSV23, but 14,953 did not. The number of patients receiving PPSV23 vaccination during specific periods is shown in Table 1. Most patients (2622 patients, 74.6%) received PPSV23 from October 2008 to December 2008. The “vaccination period” was defined as October 2008 to December 2008 to reduce the potential immortal time bias associated with the competing risk by death, that is, patients who survived long tended to be healthier than who died early, and have a greater chance of receiving vaccination. Only patients who survived to the end of the vaccination period, that is, January 1, 2009, were included. As such, 893 patients in the vaccinated group and 2960 in the unvaccinated group were further excluded from the analyses, that is, patients who died before 2009 or received PPSV23 outside the defined vaccination period (Fig. 1). The follow-up period of the vaccinated and unvaccinated groups started on January 1, 2009, and ended on the date of withdrawal from the National Health Insurance (NHI) program, death, or study termination (December 31, 2010).

**Table 1:**
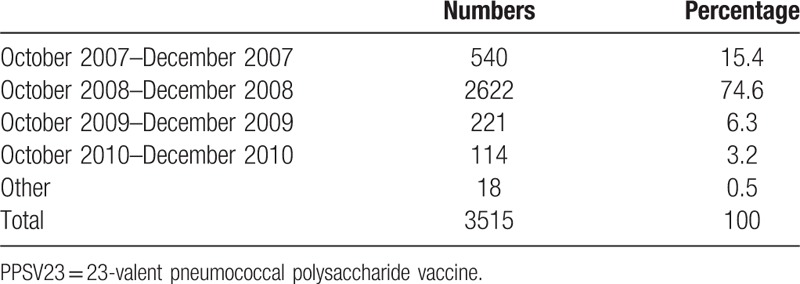
Months distribution of 23-valent pneumococcal polysaccharide vaccine vaccination in elderly patients with colorectal cancer.

Self-selection for vaccination might exist, considering the relatively low vaccination rate. Each vaccinated patient was subjected to propensity score matching to 3 unvaccinated patients to reduce potential confounding by indication that elderly people who suffered from a frequent pneumonia in the past tended to have a greater willingness to receive vaccination than the general elderly population. The propensity score was calculated from the patients’ age on January 1, 2009, sex, and number of pneumonia hospitalizations over the past 3 years. A total of 2622 vaccinated patients and 7866 unvaccinated patients were recruited (Fig. 1).

### 2.3 Measurements of endpoints and potential confounders

The primary outcome in the study was all-cause bacterial pneumonia hospitalization (*International Classification of Disease, Ninth Revision, Clinical Modification* codes for inpatient services: 481–482 and 485–486). In this study, all-cause bacterial pneumonia included invasive and noninvasive pneumonia and excluded viral pneumonia and influenza. The primary outcome was all-cause bacterial pneumonia rather than specific pneumococcal pneumonia because a definite pathogen culture result is unnecessary during pneumonia treatment. Therefore, the frequency of pneumococcal pneumonia is highly underestimated in clinical practice and in our Health Insurance Research Database (NHIRD), possibly resulting in a wrong conclusion. The potential confounders considered in this study were age, sex, influenza vaccination, vaccination period, cancer treatment modalities, comorbidity, and sociodemographic variables (Table 2). Cancer treatment modalities, including surgery, radiotherapy, chemotherapy, and targeted therapy, were also adjusted.^[4–6]^ The influenza vaccination status was also considered a potential confounder and adjusted in the analysis because most patients received PPSV23 and influenza vaccines.

**Table 2:**
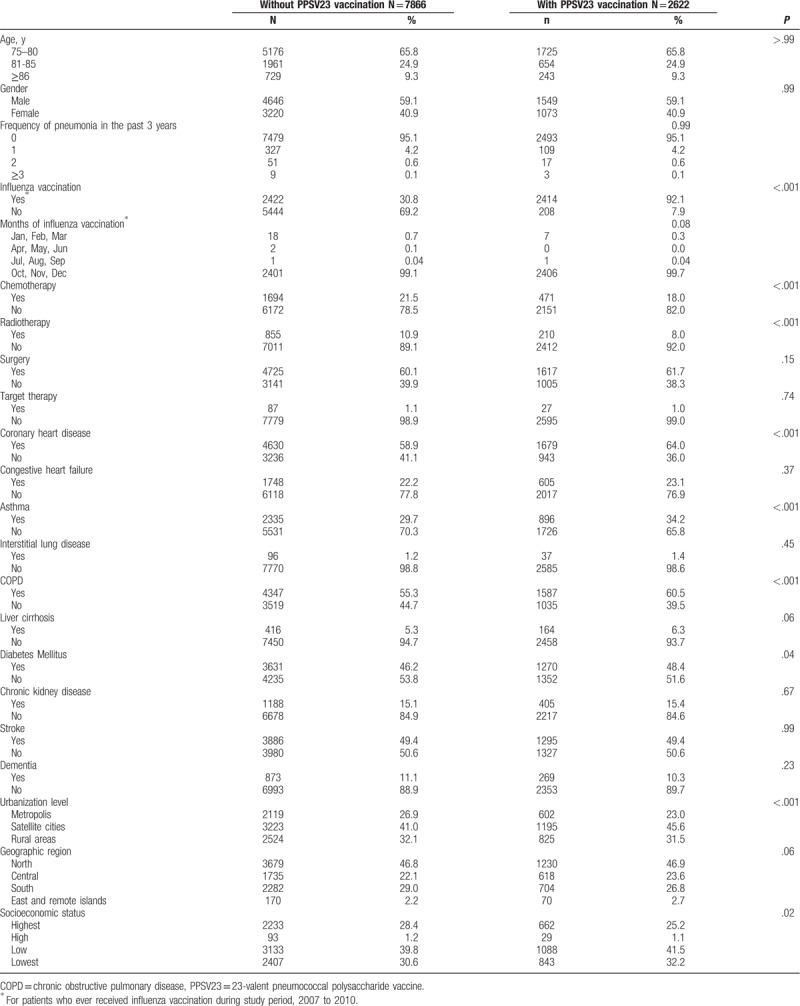
Demographic characteristics and comorbidities of elderly patients with colorectal cancer vaccinated and unvaccinated with 23-valent pneumococcal polysaccharide vaccine.

A number of major illnesses, such as coronary heart disease, congestive heart failure (CHF), asthma, interstitial lung disease, chronic obstructive pulmonary disease (COPD), liver cirrhosis, diabetes mellitus (DM), chronic kidney disease (CKD), stroke, and dementia, which could affect susceptibility to pneumonia, were included in our analysis.^[21]^ These comorbidity data were obtained from ambulatory care and inpatient hospitalization claims in 1996 to 2008.

People with higher health awareness would be more likely to be vaccinated than the general population, so several socioeconomic variables, including urbanization level, geographic region, and monthly income-based insurance premium, were also adjusted. Patients were grouped on the basis of urbanization level (i.e., urban, suburban, and rural) in accordance with the proposed classification scheme of Liu et al.^[22]^ The urbanization level was adjusted because of the distinct urban-rural difference in medical care accessibility in Taiwan.^[23]^

### 2.4 Statistical analysis

The propensity score method was used for matching. The characteristics between the 2 study groups were compared. The incidence rate of pneumonia hospitalization was calculated as the ratio of the number of pneumonia hospitalizations to the number of person-years (PYs) of follow-up. The follow-up period of both study groups started on January 1, 2009, and ended on the date of withdrawal from the NHI program, death, or study termination (December 31, 2010). The incidence rate followed a Poisson distribution, so a multivariate log-linear Poisson regression model was used to calculate the incidence rate ratios (IRRs) with all covariates included. The Kaplan-Meier method was used to estimate the overall survival time. Two statistical packages [SAS (version 9.4; SAS Institute Inc, Cary, NC) and SPSS (version 12, SPSS Inc, Chicago, IL)] were used to analyze the data. A 2-sided *P* value of <.05 was considered statistically significant.

## 3 Results

The distribution of the demographic characteristics and comorbidities, including pneumonia hospitalization history, of the 2 groups is shown in Table 2. The PPSV23-vaccinated and unvaccinated elderly patients with CRC had similar mean ± standard deviation age of 79.7 ± 3.9 and 79.8 ± 4.2 years, respectively.

A total of 1786 episodes of pneumonia hospitalization occurred in an observation period of 19,703.37 PYs in 1168 patients. The pneumonia incidence rate was lower in the vaccinated patients [85.53 per 1000 PYs; 95% confidence interval (CI): 77.41–93.65] than in the unvaccinated patients (92.38 per 1000 PYs; 95% CI: 87.47–97.28; Table 3). The proportion of the vaccinated patients with no and 1 pneumonia hospitalization per year was higher than that of the unvaccinated patients (89.1% vs 88.8%, 7.9% vs 7.2%; Table 4). The proportions of patients who had 2, 3, and >3 pneumonia hospitalizations per year were consistently lower in the vaccinated group than in the unvaccinated group (1.9% vs 2.0%, 0.5% vs 0.9%, and 0.7% vs 1.1%, respectively).

**Table 3:**
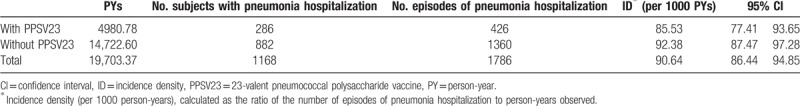
Incidence density of pneumonia hospitalization in elderly patients with colorectal cancer vaccinated and unvaccinated with 23-valent pneumococcal polysaccharide vaccine.

**Table 4:**
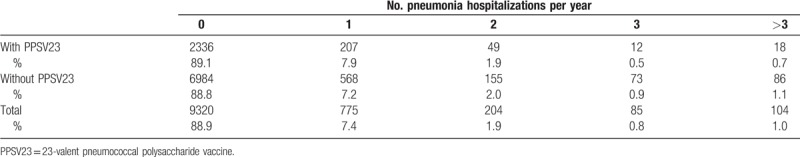
Frequency distribution of pneumonia hospitalization episodes per year in elderly patients with colorectal cancer with and without 23-valent pneumococcal polysaccharide vaccine.

After adjustment for confounders was made, our analysis showed that PPSV23 vaccination significantly reduced the pneumonia hospitalization risk, with an IRR of 0.880 (*P* = .04; Table 5). An adjusted IRR of sex significantly <1 (0.643, *P* < .001) indicated that men were more at risk of pneumonia hospitalization than women. The incidence rate of pneumonia hospitalization was increased by certain cancer treatment modalities, such as radiotherapy (adjusted IRR = 1.439, *P* < .001) and surgery (adjusted IRR = 1.158, *P* = .003), but this rate was not affected by other modalities, such as target therapy and chemotherapy.

**Table 5:**
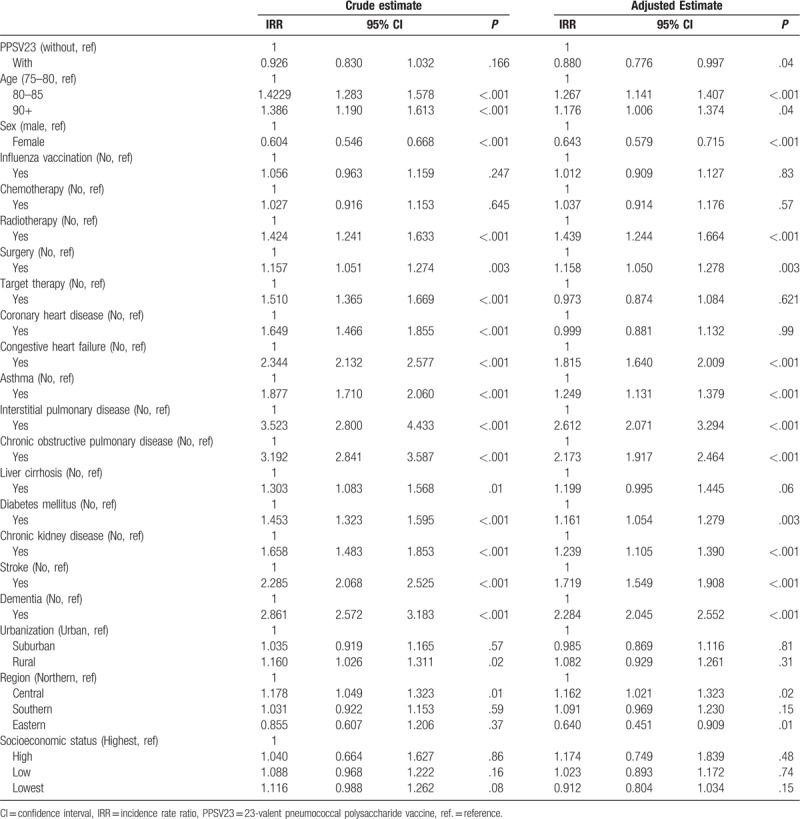
Crude and adjusted incidence rate ratio of pneumonia hospitalization in elderly patients with colorectal cancer.

PPSV23 and influenza vaccinations are administered from October to December every year in Taiwan (Table 2). PPSV23-vaccinated patients are much more likely to receive influenza vaccination than unvaccinated patients (92.1% vs 30.8% with *P* < .001; Table 2). In univariate and multivariate analyses, all covariates were, however, adjusted, and influenza vaccination had no significant effect on pneumonia hospitalization (IRR = 1.056, *P* = .247; adjusted IRR = 1.012, *P* = .83; Table 5).

The comorbidities affecting the pneumonia-hospitalization incidence rate were CHF (adjusted IRR = 1.815, *P* < .001), asthma (adjusted IRR = 1.249, *P* < .001), interstitial pulmonary disease (adjusted IRR = 2.612, *P* < .001), COPD (adjusted IRR = 2.173, *P* < .001), DM (adjusted IRR = 1.161, *P* = .003), CKD (adjusted IRR = 1.239, *P* < .001), stroke (adjusted IRR = 1.719, *P* < .001), and dementia (adjusted IRR = 2.284 *P* < .001). Sociodemographic variables, urbanization, and socioeconomic status wages did not show any significant effect on the IRR of pneumonia hospitalization. The risk of hospitalized pneumonia in the central region was higher (adjusted IRR = 1.162, *P* = .02) than that in the northern region, whereas the risk of hospitalized pneumonia in the eastern region was lower (adjusted IRR = 0.64, *P* = .01) than that in the northern region.

**Figure 2:**
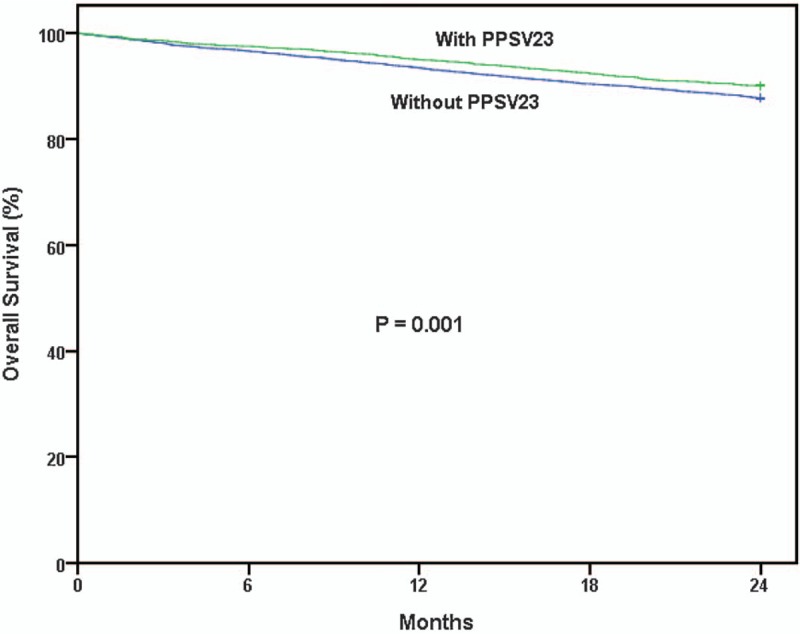
Kaplan-Meier survival curves for elderly colorectal cancer patients with and without PPSV23 vaccination (*P* = .001). Green line = with PPSV23 vaccination, blue line = without PPSV23 vaccination. PPSV23 = 23-valent pneumococcal polysaccharide vaccine.

The overall survival was significantly better in the PPSV23-vaccinated group than in the unvaccinated group (Fig. 2, *P* = .001).

## 4 Discussion

Our study indicated that pneumonia is a critical disease affecting elderly patients with CRC and aged 75 years or older. The clinical effectiveness of PPSV23 has never been studied in patients with CRC. In this population-based propensity score–matched cohort study, pneumonia hospitalization risk was decreased 12% in the vaccinated cohort. Our resulted also showed there were fewer patients in the vaccinated group with pneumonia hospitalizations ≥2 times per year, than in the unvaccinated group. In addition, vaccinated patients with CRC had higher survival rate than patients unvaccinated with PPSV23. Although anticancer therapies and aging might affect immune responses to vaccination, this study showed that elderly patients with CRC and aged 75 years or older, could benefit from PPSV23.

For the clinical benefit of PPSV23, we found that PPSV23 can significantly reduce the hospitalization frequency and mortality of patients with lung cancer during active anticancer treatment.^[24]^ Another study also showed that PPSV23 vaccination is associated with a significantly reduced rate of pneumonia hospitalization in survivors of long-term cancer.^[25]^ In our study, PPSV23 was also effective in elderly patients with CRC. It could also be considered a feasible strategy for coping with the high risk of pneumonia in elderly patients with CRC because the cost of PPSV23 is low. Our results could encourage doctors to recommend pneumococcal vaccine for patients with cancer because we approved the effectiveness of PPSV inoculation after cancer diagnosis. In clinical practice, oncologists often focus on cancer treatment and disregard the importance of pneumococcal vaccine for elderly people and patients with cancer.

The optimal timing for vaccination is an interesting question. The effectiveness of pneumococcal vaccine inoculated before cancer diagnosis is still unknown. Our PPSV-related studies on patients with lung cancer, survivors of long-term cancer, and patients with CRC have included patients who received PPSV23 after cancer diagnosis.^[24,25]^ Time interval between vaccine administration and chemotherapy initiation has, however, been rarely studied in adult patients with cancer. Choi et al^[26]^ investigated optimal vaccination timing by vaccinating patients 2 weeks before or on the day of chemotherapy initiation to determine the antibody response of patients with CRC to pneumococcal conjugate vaccine 13. They found no significant differences. Therefore, the clinical effectiveness of vaccines between different vaccine periods should be further investigated.

For the vaccinated patients, the pneumonia incidence in our study was still high possibly because of several reasons. The most important reason was that the endpoint of this study was all-cause bacterial pneumonia rather than specific pneumococcal pneumonia. The second reason was that patients were very old, that is, they were 75 years or older. The third reason was that their cancer status or cancer treatments, such as chemotherapy/radiotherapy, resulted in their relative immunosuppression status.

Influenza infection may predispose some patients to bacterial pneumonia, but influenza vaccination did not decrease the number of bacterial pneumonia hospitalizations in this study possibly because of the following: our endpoint outcome was strictly bacterial pneumonia, not viral pneumonia, and influenza; some circulating virus strains were covered by the influenza vaccine in that year; and our endpoint was hospitalized pneumonia, which is more severe than CAP.

In our study, surgery included laparoscopic and open surgery. Surgery and radiotherapy were associated with a high risk of pneumonia hospitalization in elderly patients with CRC. Many certain comorbidities, such as CHF, asthma, interstitial pulmonary disease, COPD, DM, CKD, stroke, and dementia, increased the risk of pneumonia hospitalization in this study. Jackson et al^[27]^ identified CHF, asthma, COPD, DM, stroke, dementia, and lung cancer as risk factors of pneumonia in general people aged 65 years or older; they also identified CHF, asthma, COPD, and dementia as risk factors in elderly patients with cancer. In a multicenter and retrospective cohort study in South Korea, old age, more comorbidities, ulcer disease, history of pneumonia, and smoking are associated with an increased incidence of pneumonia within 1 year after cancer surgery.^[28]^

### 4.1 Study strengths

This study had several strengths. First, it was a nationwide population-based study that included all patients with CRC and all hospitals in Taiwan, leaving a low chance of selection bias and attrition bias (loss to follow-up) and having a relatively large sample size. Second, the utilization of propensity score matching strategy, with age, sex, and previous personal pneumonia history, to select unvaccinated patients also helped reduce confounding by indication, that is, elderly people who suffered from frequent pneumonia would have a greater willingness to receive vaccination than the general elderly population. Lastly, a PYs approach was used to determine the occurrence of multiple pneumonia incidence, thereby reducing the potential bias due to different lengths of follow-up between vaccinated and unvaccinated groups. This finding was important because of the relatively short life expectancy of elderly patients with CRC (age older than 75 years).

### 4.2 Study limitations

Our study also had several limitations. First, we conducted an observational nationwide population-based matched cohort study rather than a randomized trial, so our study was still exposed to certain unmeasured confounders, even though our patients were matched with propensity score and analyzed through multivariate analysis. Second, this study did not collect cancer stage information. Cancer treatment modalities, such as surgery, chemotherapy, radiotherapy, and target therapy, which are, however, relevant to individual cancer stage, were included in our analysis. The PYs approach eliminated the effect of different lengths of follow-up due to different cancer stages. Third, the database used was limited to routinely collected data for the National Health Insurance system, that is, it does not include nonroutinely collected data, such as smoking personal history, although COPD was included in the adjustment in this study. Fourth, the conclusion of this population-based cohort study was limited to the patients with CRC of this age group because the “free vaccine” policy applies only to those older than 75 years.

## 5 Conclusion

PPSV23 vaccination was associated with a significantly reduced rate of pneumonia hospitalization in elderly patients with CRC.

## Author contributions

**Conceptualization:** Wen-Yen Chiou.

**Data curation:** Feng-Chun Hsu.

**Formal analysis:** Wen-Yen Chiou, Liang-Cheng Chen, Feng-Chun Hsu, Shiang-Jiun Tsai, Ben-Hui Yu.

**Funding acquisition:** Shih-Kai Hung, Moon-Sing Lee.

**Investigation:** Shih-Kai Hung, Moon-Sing Lee.

**Methodology:** Chung-Yi Li.

**Project administration:** Feng-Chun Hsu, Moon-Sing Lee.

**Resources:** Shih-Kai Hung, Hon-Yi Lin, Moon-Sing Lee.

**Software:** Chung-Yi Li.

**Supervision:** Shih-Kai Hung, Hon-Yi Lin, Moon-Sing Lee, Chung-Yi Li.

**Validation:** Hon-Yi Lin, Liang-Cheng Chen, Moon-Sing Lee.

**Writing – original draft:** Wen-Yen Chiou.

**Writing – review and editing:** Hon-Yi Lin, Moon-Sing Lee, Chung-Yi Li.
